# Supplementary material for: A modular framework for multiscale, multicellular, spatiotemporal modeling of acute primary viral infection and immune response in epithelial tissues and its application to drug therapy timing and effectiveness: A multiscale model of viral infection in epithelial tissues
Source: bioRxiv. 2020 Sep 26:2020.04.27.064139. Preprint. [Version 4] doi: 10.1101/2020.04.27.064139 (PMC7263495; doi:10.1101/2020.04.27.064139)
Supplement: 1 [file NIHPP2020.04.27.064139-supplement-1.pdf]

bioRxiv preprint doi: https://doi.org/10.1101/2020.04.27.064139. this version posted September 26, 2020. The copyright holder for this preprint (which was not certified by peer review) is the author/funder. It is made available under a CC-BY 4.0 International license.

Supplementary Materials

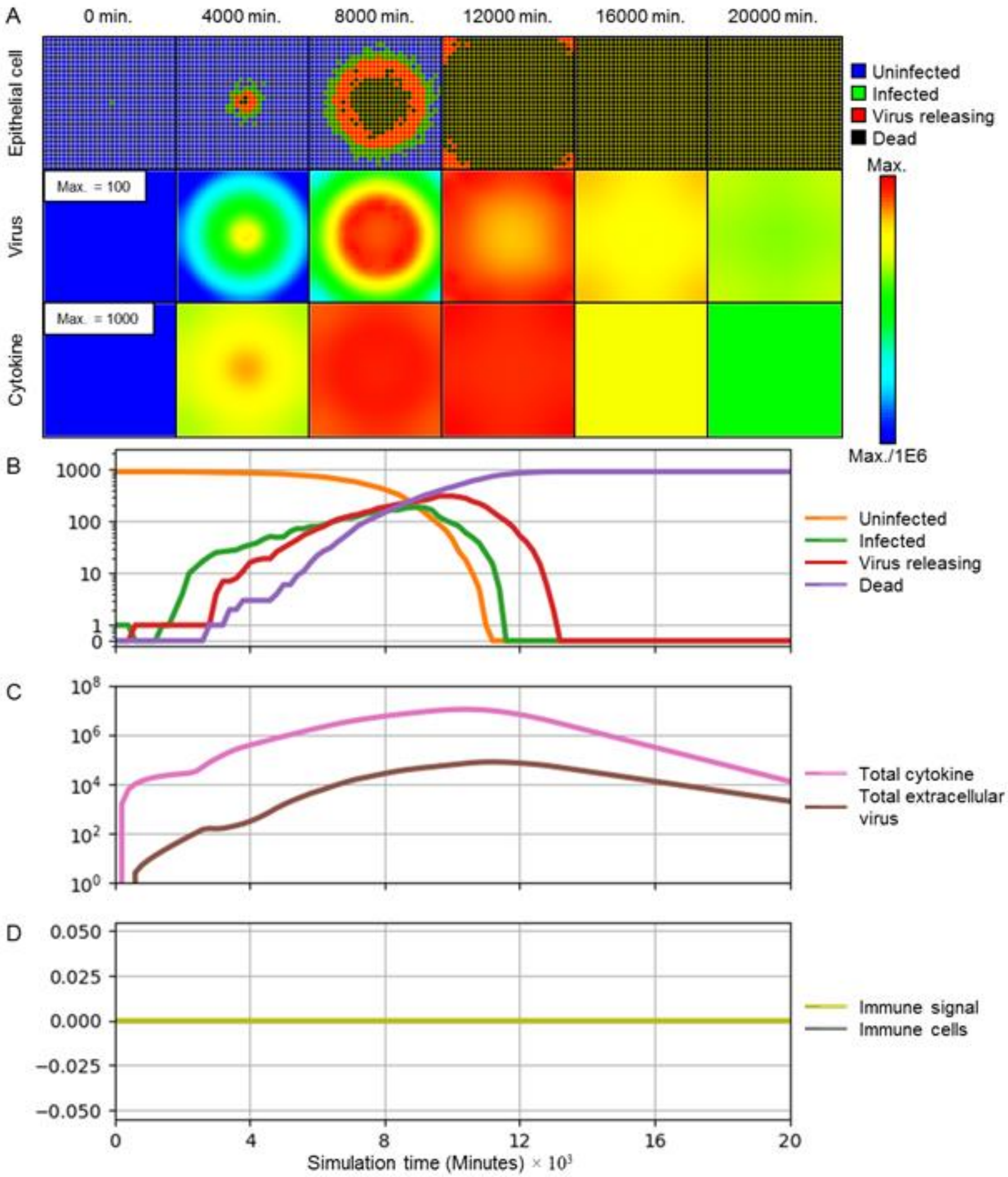

Fig S1. No immune system.

Simulation of the progression of infection in a patch of epithelial tissue, with all parameters as in Fig 3, but with no cellular immune system response corresponding to virus spread in an *in vitro* or organoid culture, or a severely immunosuppressed individual. (A) Snapshots of spatial configuration *vs* time showing progression of simulated

bioRxiv preprint doi: https://doi.org/10.1101/2020.04.27.064139. this version posted September 26, 2020. The copyright holder for this preprint (which was not certified by peer review) is the author/funder. It is made available under a CC-BY 4.0 International license.

infection. Columns, left to right: 0 minutes (time of initial infection), 4000 minutes (67 hours, 2 ¾ days) after infection, 8000 minutes (133 hours, 5 ½ days), 12000 minutes (200 hours, 8 ⅓ days), 16000 minutes (267 hours, 11 days), and 20000 minutes (333 hours, 14 days). First row: epithelial cell layer composed of uninfected (blue), infected (green), virus releasing (red) cells and dead cells (black). Second row: level of extracellular virus field. Third row: extracellular cytokine field. Fields are color-coded on a logarithmic scale: red corresponds to the chosen maximum value specified in the first panel, blue to six orders of magnitude lower than the maximum value, and values outside this range are colored as their closest border value. (B-D) Simulation time series. (B) Number of uninfected (orange), infected (green), virus releasing (red) and dead (purple) epithelial cells on a logarithmic scale *vs* time *vs* time in minutes. (C) Total extracellular cytokine (magenta) and total extracellular virus (brown) on a logarithmic scale *vs* time in minutes. (D) Value of the immune recruitment signal S (yellow) and number of immune cells (grey) on a linear scale *vs* time in minutes.

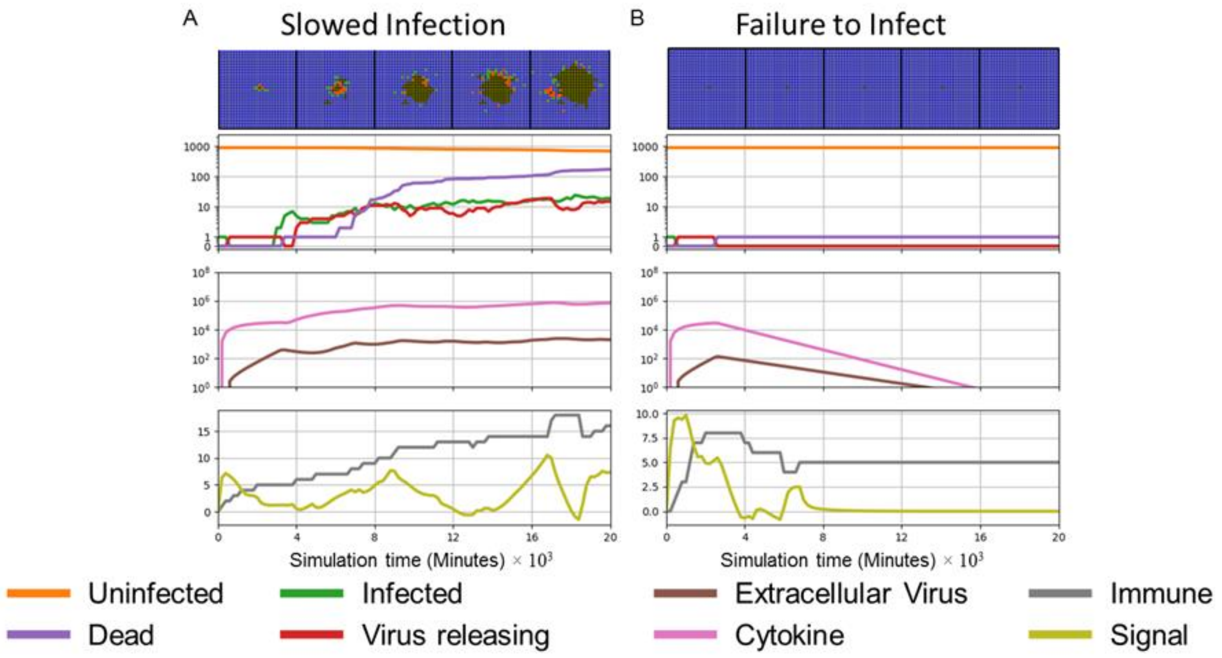

Fig S2. Special cases of spatiotemporal infection dynamics.

(A) A border case of slowed infection towards containment is Slowed Infection with constant virus: when the net effectiveness of viral and immune dynamics are balanced, the number of infected cells and the total extracellular virus fluctuate around steady state levels. (B) A limit case of Clearance is the failure to infect: initially infected cells may replicate and secrete virus, but insufficiently so to infect other cells during simulation time such that any initially infected cells vanish and total extracellular virus drops below a threshold of 10^{-3} per cell area.

bioRxiv preprint doi: https://doi.org/10.1101/2020.04.27.064139. this version posted September 26, 2020. The copyright holder for this preprint (which was not certified by peer review) is the author/funder. It is made available under a CC-BY 4.0 International license.

Table S1. Varying parameters in simulations shown in Fig 4. Virus-receptor association affinity and immune response delay coefficient shown for no immune response (Fig 4A), widespread infection (Fig 4B), slowed infection (Fig 4C), containment (Fig 4D), recurrence (Fig 4E) and clearance (Fig 4F). All other parameters are as in Table 1.

Parameter	No immune response	Widespread infection	Slowed infection	Containment	Recurrence	Clearance
Virus-receptor association affinity k_{on} (M^{-1} s^{-1})	1.4×10^4	1.4×10^4	1.4×10^3	1.4×10^2	1.4×10^6	1.4×10^4
Immune response delay coefficient β_{delay} (s)	-	1.2×10^7 s	1.2×10^8 s	1.2×10^5 s	1.2×10^4 s	1.2×10^5 s

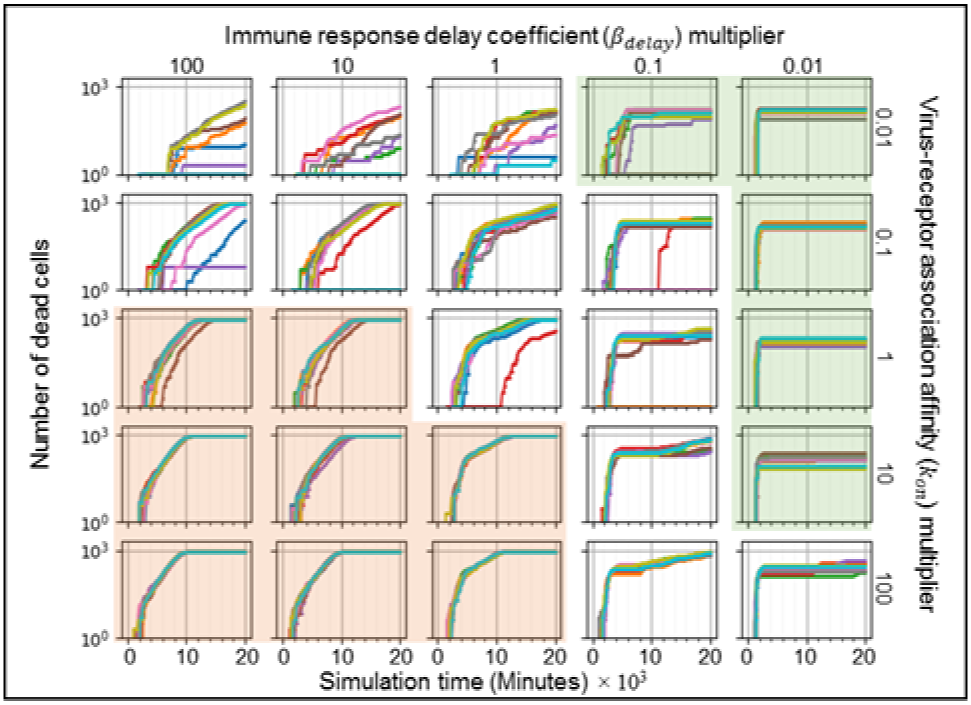

Fig S3. Time series of the number of uninfected cells for simulations in Fig 5.

Logarithmic multidimensional parameter sweep performed by running 10 simulation replicas increasing and decreasing the baseline parameter values 10-fold and 100-fold for all parameter sets and replicas in Fig 5. Results show consistent containment/clearance for small k_{on} and small β_{delay} (upper right, green-shaded subplots), widespread infection for high k_{on} and small β_{delay} (lower left, orange-shaded subplots), and multiple outcomes for the same parameter values (uncolored subplots). Number of cells are shown on a logarithmic scale *vs* time in minutes.

bioRxiv preprint doi: https://doi.org/10.1101/2020.04.27.064139. this version posted September 26, 2020. The copyright holder for this preprint (which was not certified by peer review) is the author/funder. It is made available under a CC-BY 4.0 International license.

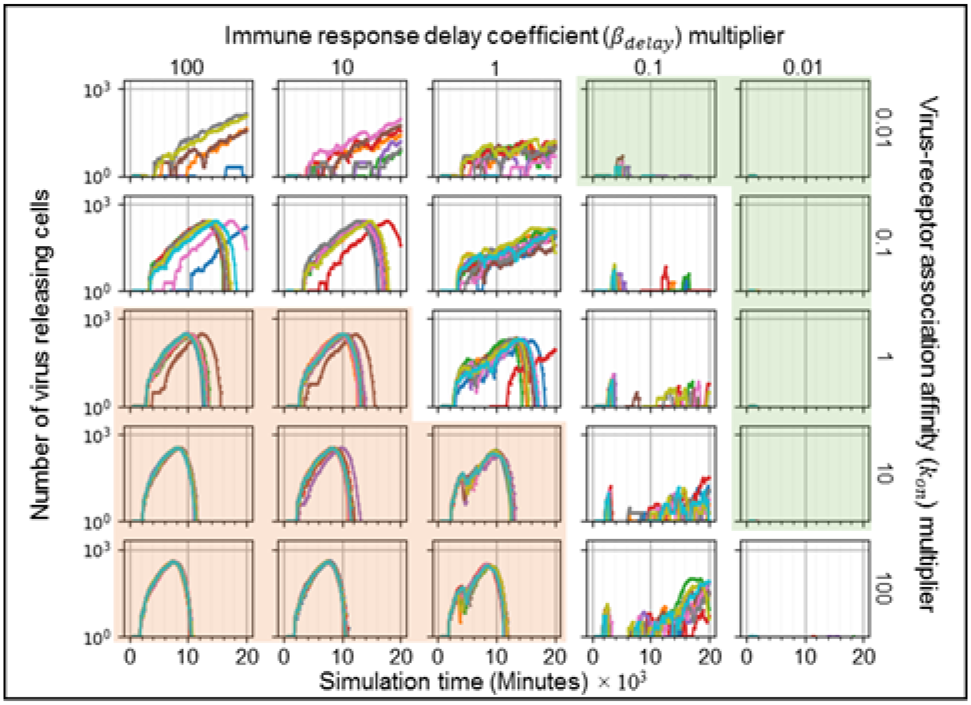

Fig S4. Time series of the number of virus releasing cells for simulations in Fig 5.

Logarithmic multidimensional parameter sweep performed by running 10 simulation replicas increasing and decreasing the baseline parameter values 10-fold and 100-fold for all parameter sets and replicas in Fig 5. Results show consistent containment/clearance for small k_{on} and small β_{delay} (upper right, green-shaded subplots), widespread infection for high k_{on} and small β_{delay} (lower left, orange-shaded subplots), and multiple outcomes for the same parameter values (uncolored subplots). Number of cells are shown on a logarithmic scale *vs* time in minutes.

bioRxiv preprint doi: https://doi.org/10.1101/2020.04.27.064139. this version posted September 26, 2020. The copyright holder for this preprint (which was not certified by peer review) is the author/funder. It is made available under a CC-BY 4.0 International license.

1865 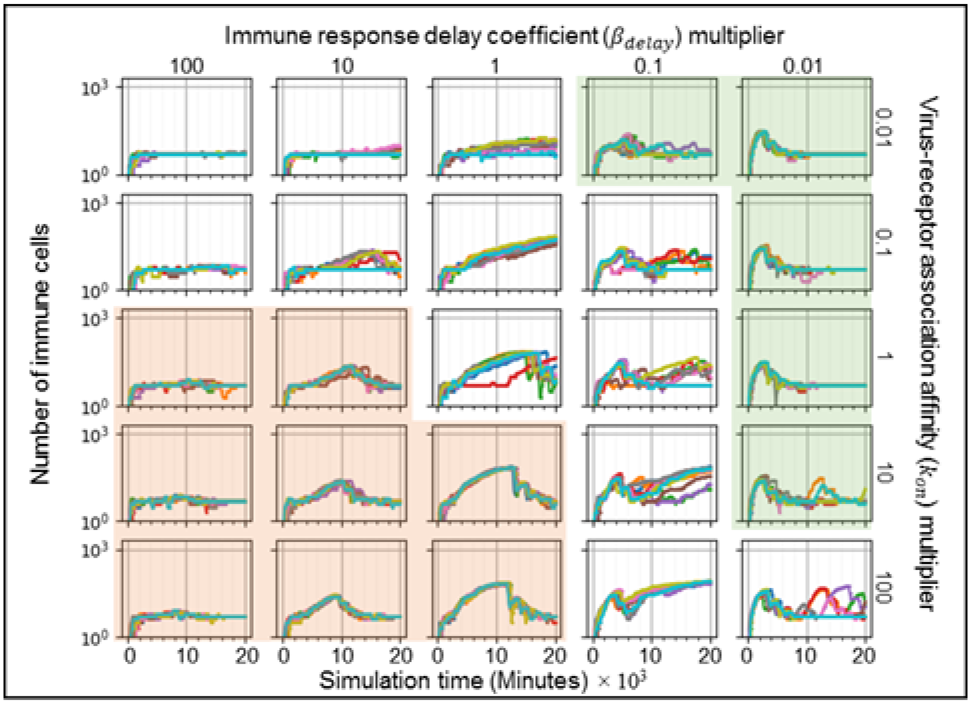

1866 **Fig S5. Time series of the number of immune cells for simulations in Fig 5**.

1867 Logarithmic multidimensional parameter sweep performed by running 10 simulation replicas increasing and decreasing
1868 the baseline parameter values 10-fold and 100-fold for all parameter sets and replicas in Fig 5. Results show consistent
1869 containment/clearance for small k_{on} and small β_{delay} (upper right, green-shaded subplots), widespread infection for
1870 high k_{on} and small β_{delay} (lower left, orange-shaded subplots), and multiple outcomes for the same parameter values
1871 (uncolored subplots). Number of cells are shown on a logarithmic scale *vs* time in minutes.

1872

bioRxiv preprint doi: https://doi.org/10.1101/2020.04.27.064139. this version posted September 26, 2020. The copyright holder for this preprint (which was not certified by peer review) is the author/funder. It is made available under a CC-BY 4.0 International license.

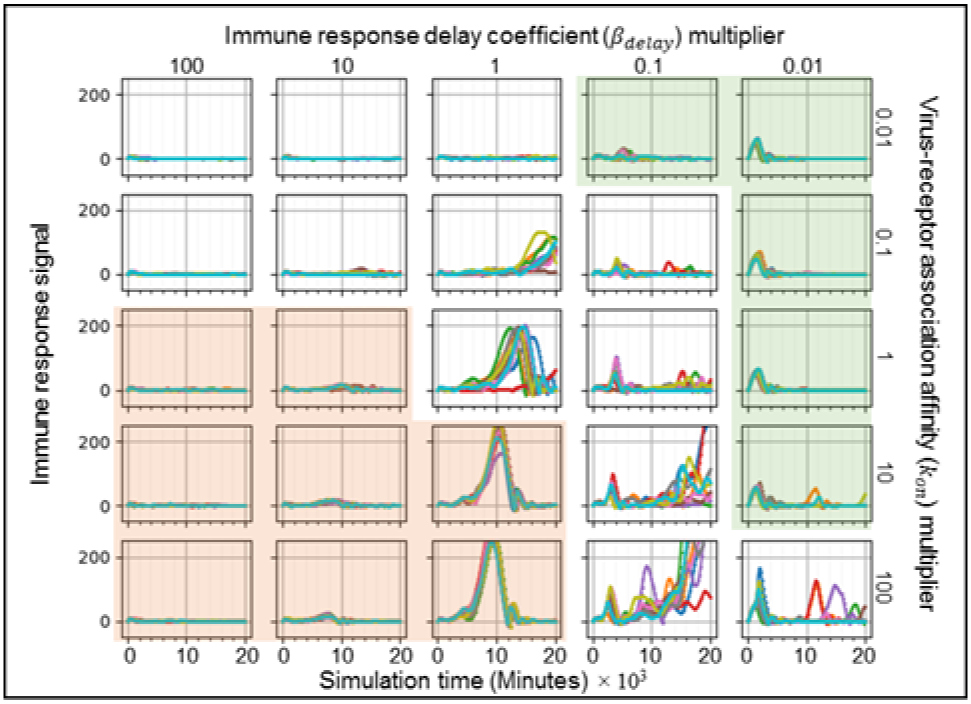

Fig S6. Time series of the immune response state variable S for simulations in Fig 5.

Logarithmic multidimensional parameter sweep performed by running 10 simulation replicas increasing and decreasing the baseline parameter values 10-fold and 100-fold for all parameter sets and replicas in Fig 5. Results show consistent containment/clearance for small k_{on} and small β_{delay} (upper right, green-shaded subplots), widespread infection for high k_{on} and small β_{delay} (lower left, orange-shaded subplots), and multiple outcomes for some parameter sets (unshaded subplots). S is shown on a linear scale *vs* time in minutes.

bioRxiv preprint doi: https://doi.org/10.1101/2020.04.27.064139. this version posted September 26, 2020. The copyright holder for this preprint (which was not certified by peer review) is the author/funder. It is made available under a CC-BY 4.0 International license.

1881 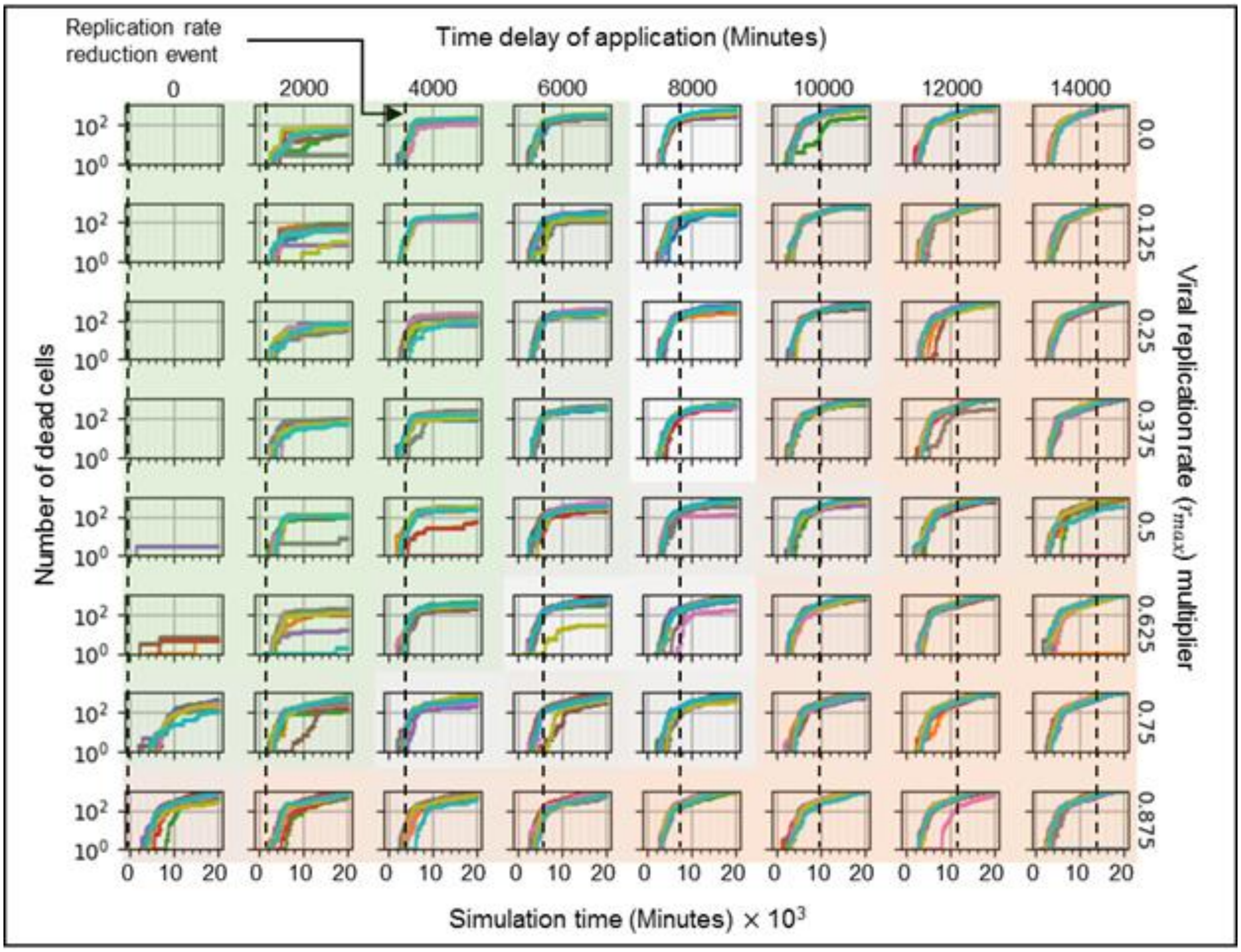

1882 **Fig S7. Simulations from Fig 8, showing the number of dead cells.**

1883 Time series of the number of dead cells for each simulation replica in Fig 8. Number of cells is shown on a logarithmic

1884 scale *vs* time in minutes.

1885

bioRxiv preprint doi: https://doi.org/10.1101/2020.04.27.064139. this version posted September 26, 2020. The copyright holder for this preprint (which was not certified by peer review) is the author/funder. It is made available under a CC-BY 4.0 International license.

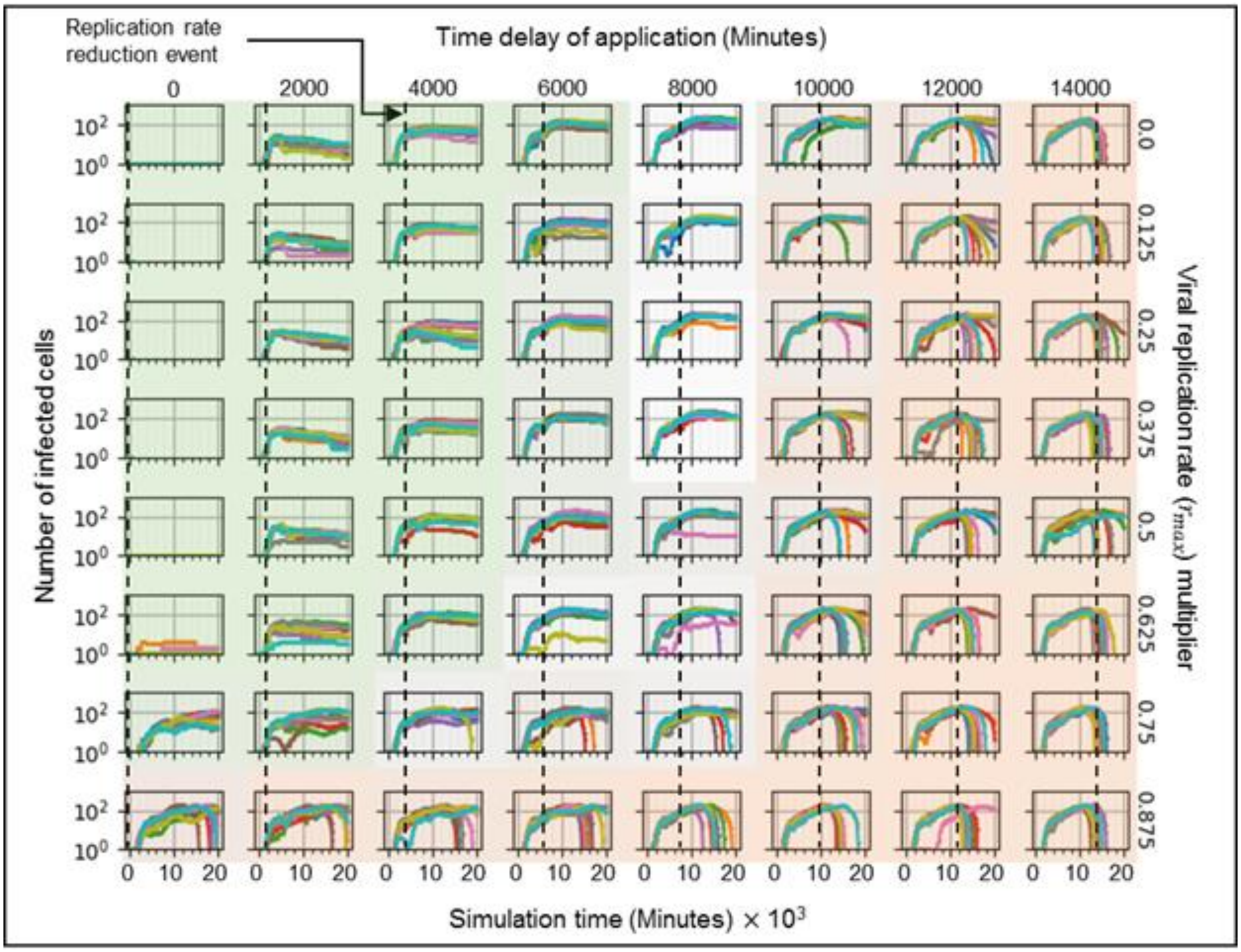

Fig S8. Simulations from Fig 8, showing the number of infected cells.

Time series of the number of infected cells for each simulation replica in Fig 8. Number of cells is shown on a logarithmic scale *vs* time in minutes.

bioRxiv preprint doi: https://doi.org/10.1101/2020.04.27.064139. this version posted September 26, 2020. The copyright holder for this preprint (which was not certified by peer review) is the author/funder. It is made available under a CC-BY 4.0 International license.

1891 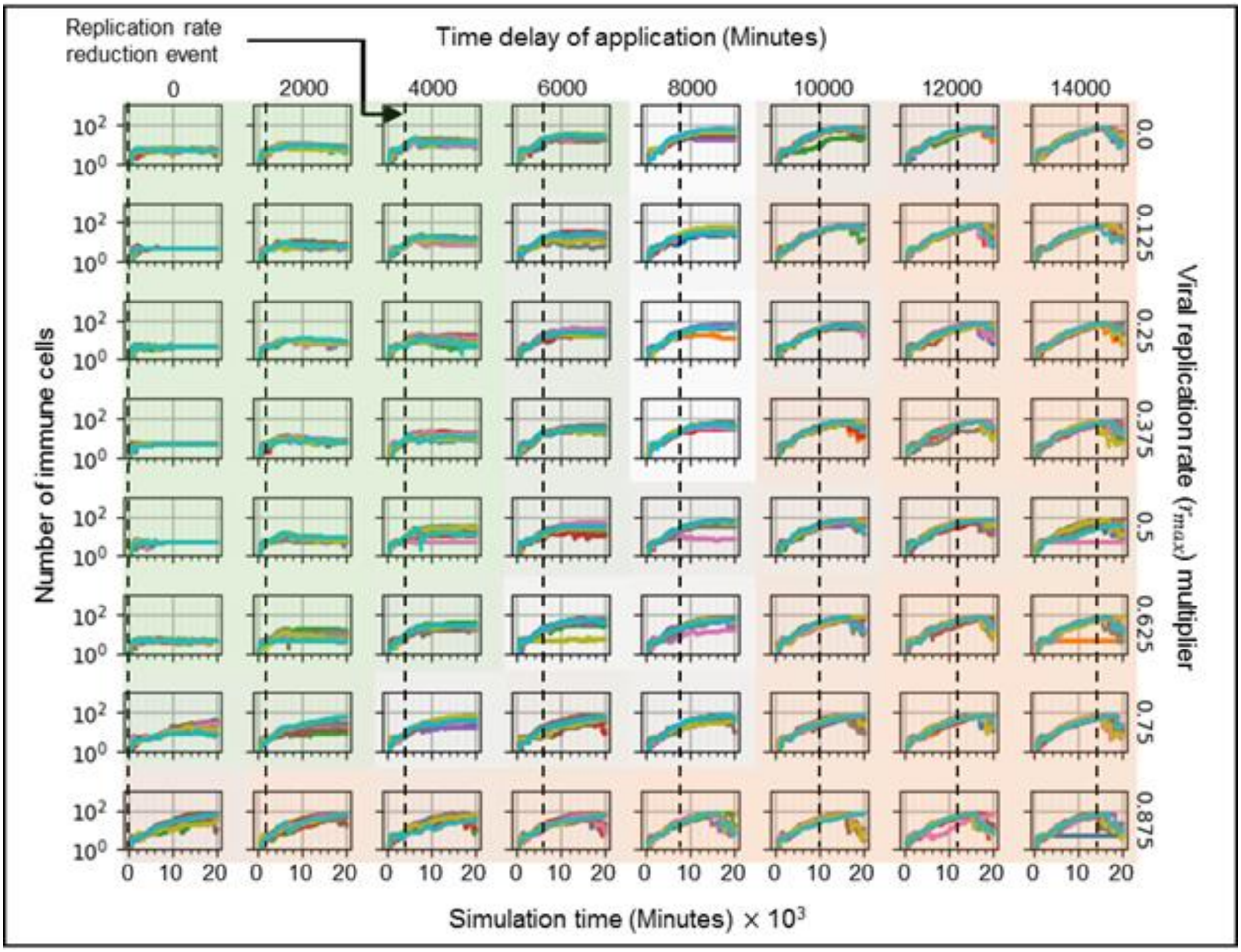

1892 **Fig S9. Simulations from Fig 8 showing the number of immune cells.**

1893 Time series of the number of immune cells for each simulation replica in Fig 8. Number of cells is shown on a logarithmic

1894 scale *vs* time in minutes.

1895

bioRxiv preprint doi: https://doi.org/10.1101/2020.04.27.064139. this version posted September 26, 2020. The copyright holder for this preprint (which was not certified by peer review) is the author/funder. It is made available under a CC-BY 4.0 International license.

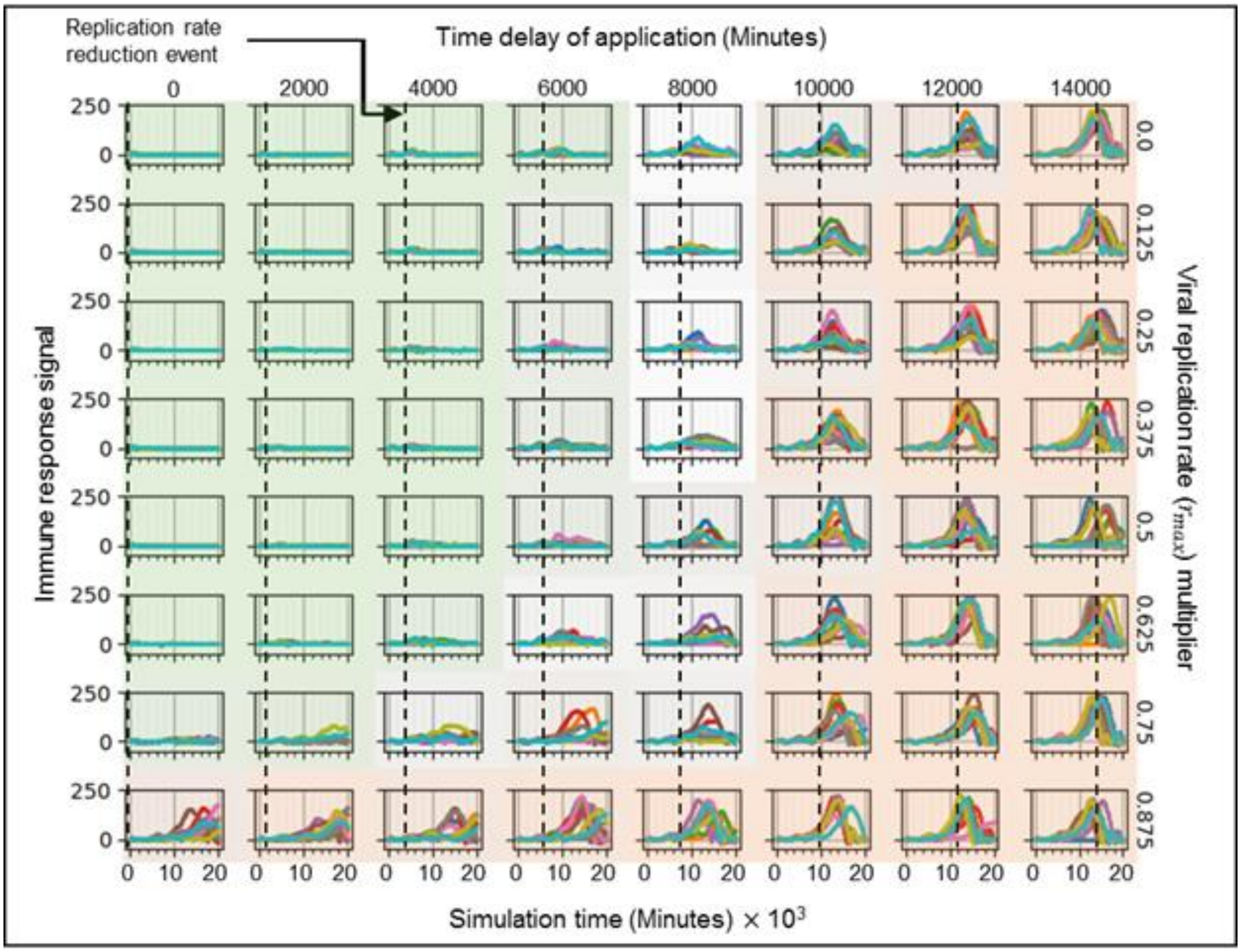

Fig S10. Simulations from Fig 8 showing the immune response state variable.

Time series of the immune response state variable S for each simulation replica in Fig 8. S is shown on a linear scale *vs* time in minutes.

bioRxiv preprint doi: https://doi.org/10.1101/2020.04.27.064139. this version posted September 26, 2020. The copyright holder for this preprint (which was not certified by peer review) is the author/funder. It is made available under a CC-BY 4.0 International license.

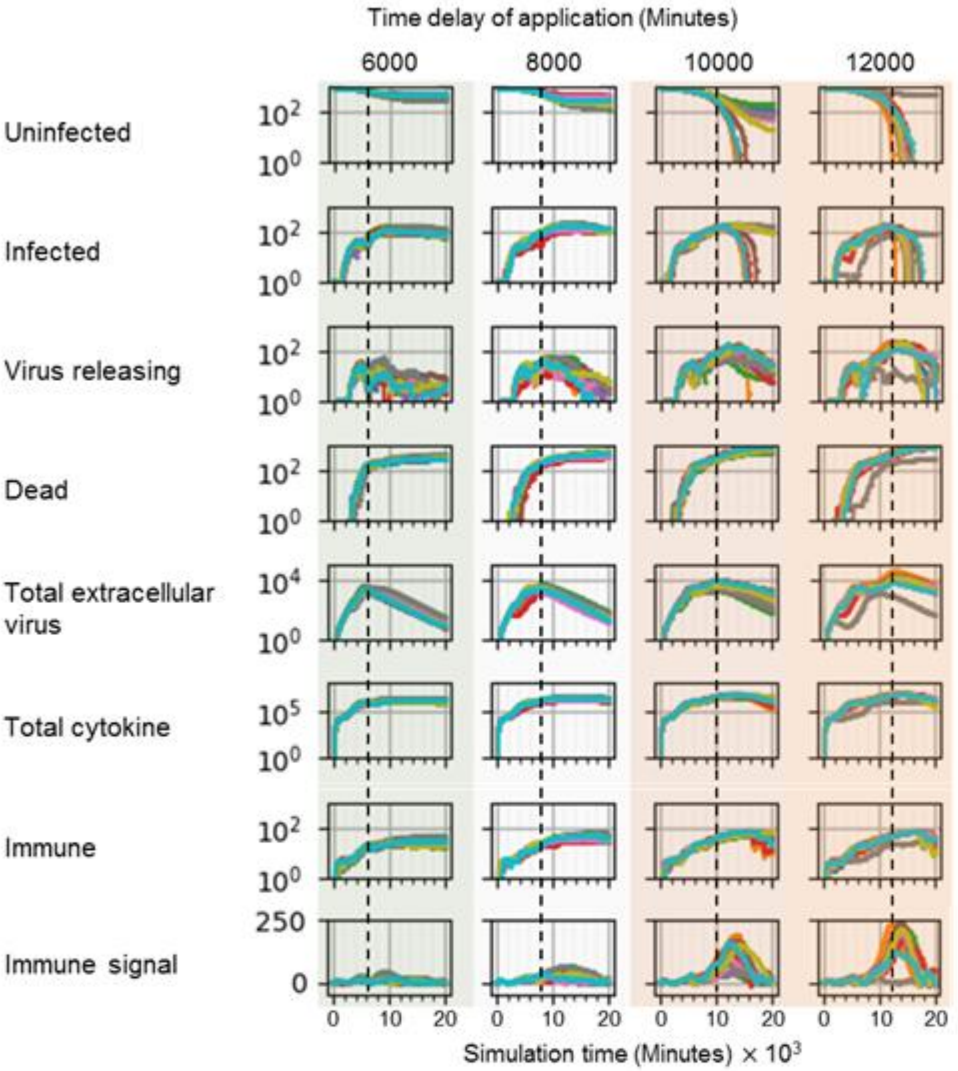

Fig S11. Variation in time of first treatment after infection with a reduced viral RNA replication rate causes a bifurcation in simulation outcomes.

Simulations and parameters are as in Figs 9-11, for a viral replication rate multiplier of 0.375 and, from left to right, time delays of application of 6000, 8000, 10000, and 12000 minutes (100, 133, 167 and 200 hours, 4, 5 ½, 7 and 8 ⅓ days) (dashed lines). Results from all simulation replicas are shown vs time in minutes for, from top to bottom: number of uninfected cells, number of infected cells, number of virus releasing cells, number of dead cells, total extracellular virus, total cytokine, number of immune cells, and immune response state variable S. Parameter set subplots are shaded as in Figs 9-11 according to simulation outcomes.

bioRxiv preprint doi: https://doi.org/10.1101/2020.04.27.064139. this version posted September 26, 2020. The copyright holder for this preprint (which was not certified by peer review) is the author/funder. It is made available under a CC-BY 4.0 International license.

Downloading and running the simulation

The COVID-19 simulation's source code is available in the GitHub repository https://github.com/covid-tissue-models/covid-tissue-response-models/tree/master/CC3D/Models/BiocIU/SARSCoV2MultiscaleVTM. The simulation is a model specification which runs in the CompuCell3D virtual-tissue simulation environment. To run the simulation requires installation of CompuCell3D version 4.1.1 or later. CompuCell3D is open-source and runs on Windows, Mac and Linux operating systems. It can be downloaded from https://compucell3d.org/SrcBin. Installers are available for Windows operating systems and Mac installation also does not require compilation. CompuCell3D's manuals are available at https://compucell3d.org/Manuals. The COVID-19 simulation can also be run online without requiring any installations or downloads on the nanoHUB servers at https://nanohub.org/tools/cc3dcovid19/. Use of nanoHUB is free but requires user registration. The simulation may take a few moments to load in its nanoHUB deployment; during load the simulation area will be blue.

Twedit++ is a specialized text editor for CompuCell3D simulations which comes packaged with CompuCell3D. Tweedit++ can open *cc3d* file extensions which contain the simulation file structure for CompuCell3D simulations. To open the COVID-19 simulation click "Open CC3D Project" (Fig S12) and select *ViralInfectionVTM.cc3d* in <repository-folder>/covid-tissue-response-models/CC3D/Models/BiocIU/SARSCoV2MultiscaleVTM/Model. Once opened, *ViralInfectionVTM.cc3d* will appear in the left- hand panel "CC3D Simulation" (Fig S13). Double click on it to open all simulation files. The main simulation files are: *ViralInfectionVTM.xml*, which defines certain simulation properties (*e.g.*, cell types, lattice

bioRxiv preprint doi: https://doi.org/10.1101/2020.04.27.064139. this version posted September 26, 2020. The copyright holder for this preprint (which was not certified by peer review) is the author/funder. It is made available under a CC-BY 4.0 International license.

size, energy-constraint plugins, diffusive fields); *ViralInfectionVTMSteppables.py*, which defines the simulation's initial conditions, main interactions and dynamics (*e.g.*, cell initialization, immune-cell recruitment, secretion by cells into fields, uptake by cells from fields); *ViralInfectionVTMSteppableBasePy.py*, where the viral infection Antimony submodel is declared; *ViralInfectionVTMModelInputs.py*, which sets the parameters. The submodels in *ViralInfectionVTMSteppables.py* are organized as python classes, making them easy to modify. Tweedit++ can also copy and rename the simulation project to a new directory by using `CC3D Project; Save Project As`. However the save as does not copy the folder `<...>/Model/nCoVToolkit`, which must be copied into the new simulation directory separately.

CompuCell3D *Player* is a GUI tool which executes CompuCell3D simulations during desktop execution (or on nanoHUB). In order to run the simulation either right click *ViralInfectionVTM.cc3d* in the left hand panel and select "Open In Player" or open *CompuCell3D.exe* to open the CompuCell3D Player and select `File; Open Simulation File` and open *ViralInfectionVTM.cc3d*. Once the simulation is open in Player click play (on the nanoHub deployment the simulation should start automatically). Player will display windows with the cell lattice rendered (Fig S14), set the z-plane to 0 to visualize the epithelial cells and the z-plane to 1 to visualize the immune cells. More windows can be created (menu `Window; New graphics Window,` Fig S15) as needed to visualize the virus, cytokine, oxidative agent fields. Each window has a drop-down menu containing the selection of fields that can be rendered (*i.e.*, the chemical fields and the cell field, Fig S16).

bioRxiv preprint doi: https://doi.org/10.1101/2020.04.27.064139. this version posted September 26, 2020. The copyright holder for this preprint (which was not certified by peer review) is the author/funder. It is made available under a CC-BY 4.0 International license.

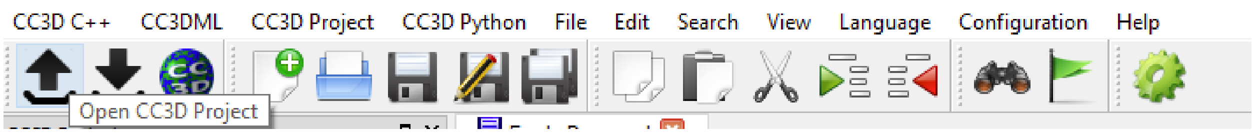

Fig S12. Opening a CompuCell3D project in Tweedit++.

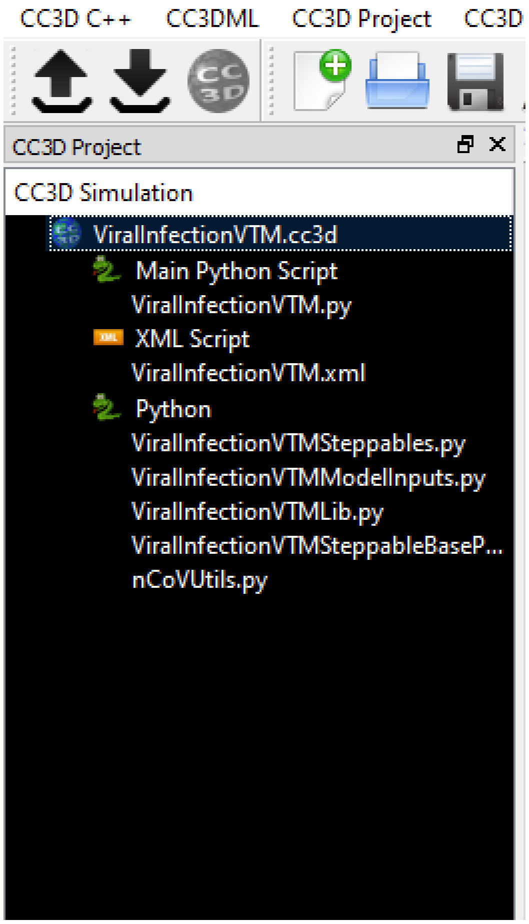

Fig S13. Tweedit++'s left hand panel with simulation project files opened.

bioRxiv preprint doi: https://doi.org/10.1101/2020.04.27.064139. this version posted September 26, 2020. The copyright holder for this preprint (which was not certified by peer review) is the author/funder. It is made available under a CC-BY 4.0 International license.

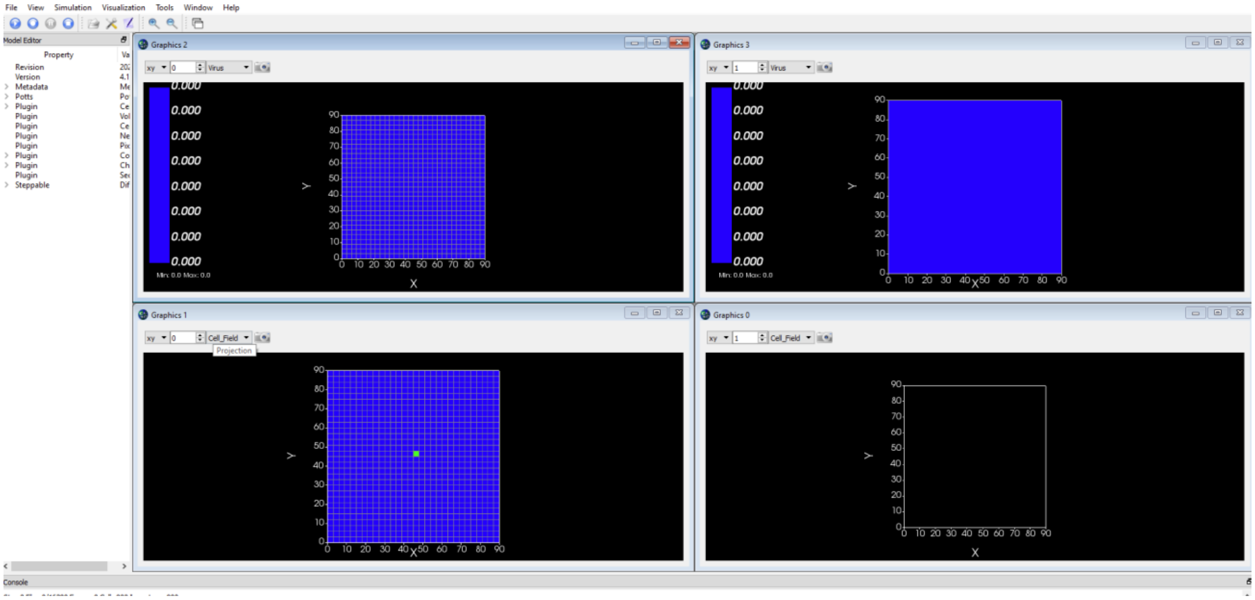

Fig S14. Example of CompuCell3D's player open with the COVID-19 simulation loaded.

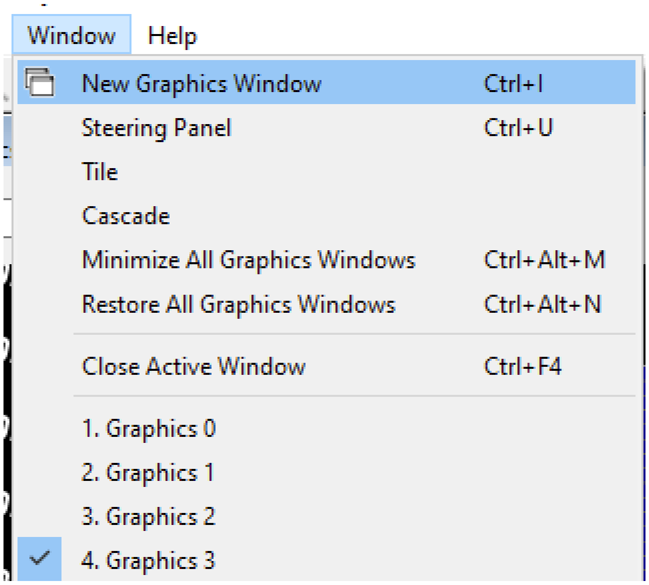

Fig S15. How to open a new simulation render window in CompuCell3D Player.

bioRxiv preprint doi: https://doi.org/10.1101/2020.04.27.064139. this version posted September 26, 2020. The copyright holder for this preprint (which was not certified by peer review) is the author/funder. It is made available under a CC-BY 4.0 International license.

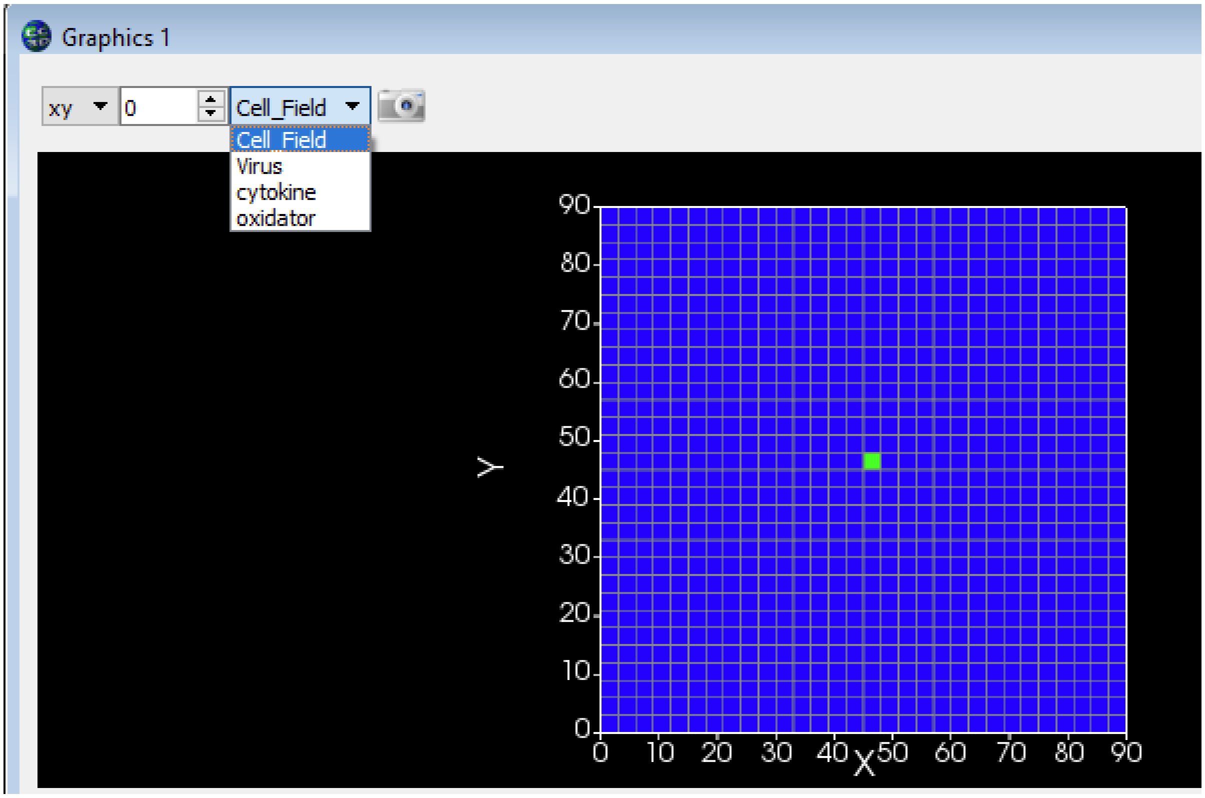

Fig S16. Drop-down menu in simulation render window to select which field to render.

bioRxiv preprint doi: https://doi.org/10.1101/2020.04.27.064139. this version posted September 26, 2020. The copyright holder for this preprint (which was not certified by peer review) is the author/funder. It is made available under a CC-BY 4.0 International license.

Sensitivity analysis of the baseline parameter set

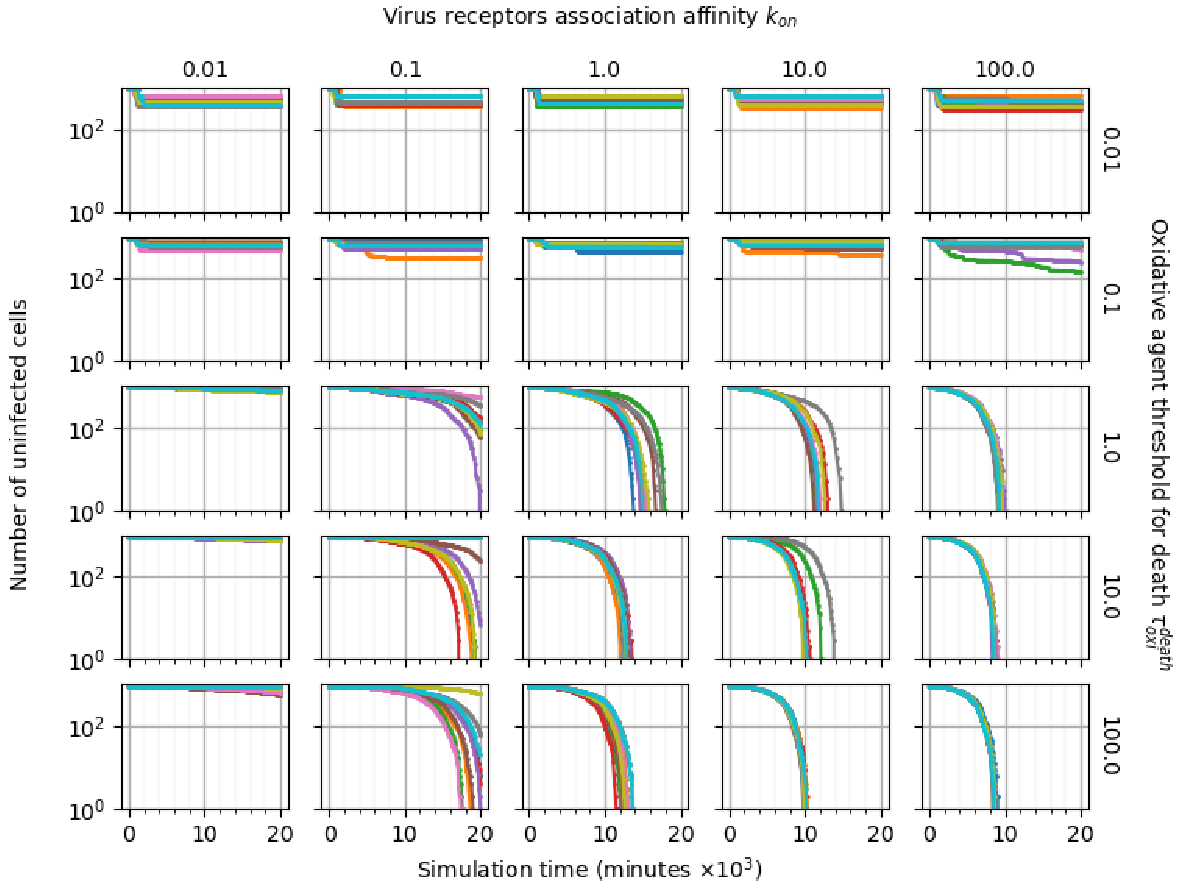

Fig S17. Pairwise parameter sweep of the oxidative agent threshold for death τ_{oxi}^{death} and the virus-receptor association affinity k_{on} (× 0.01,× 0.1,× 1,× 10,× 100) around their baseline values, with ten simulation replicas per parameter set (all other parameters have their baseline values as given in Table 1). The number of uninfected epithelial cells for each simulation replica for each parameter set, plotted on a logarithmic scale, *vs* time displayed in minutes.

bioRxiv preprint doi: https://doi.org/10.1101/2020.04.27.064139. this version posted September 26, 2020. The copyright holder for this preprint
(which was not certified by peer review) is the author/funder. It is made available under a CC-BY 4.0 International license.

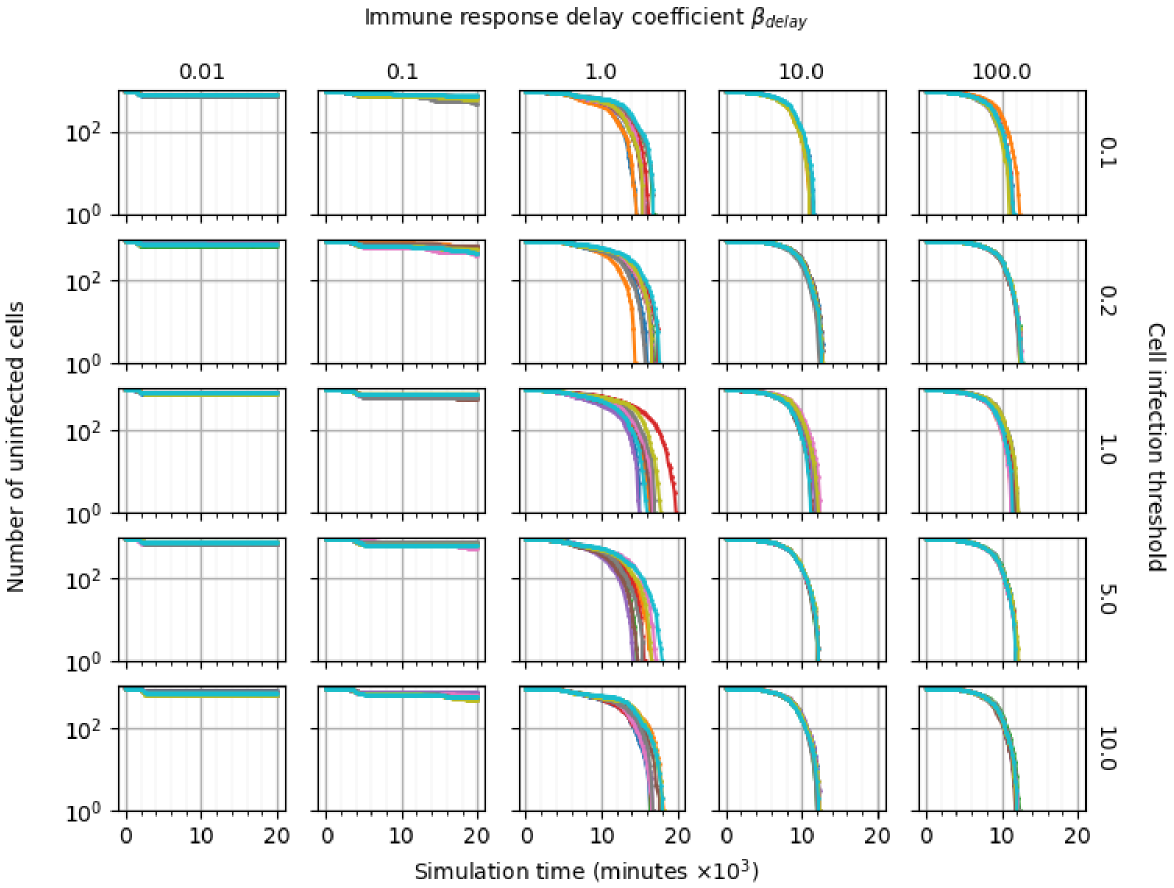

Fig S18. Pairwise parameter sweep of the immune response delay β_{delay} ($\times 0.01, \times\ 0.1, \times\ 1, \times\ 10, \times\ 100$) and infection threshold ($\times 0.1, \times\ 0.2, \times\ 1, \times\ 5, \times\ 10$) around their baseline values, with ten simulation replicas per parameter set (all other parameters have their baseline values as given in Table 1).

The number of uninfected epithelial cells for each simulation replica for each parameter set, plotted on a logarithmic scale, *vs* time displayed in minutes.

bioRxiv preprint doi: https://doi.org/10.1101/2020.04.27.064139. this version posted September 26, 2020. The copyright holder for this preprint (which was not certified by peer review) is the author/funder. It is made available under a CC-BY 4.0 International license.

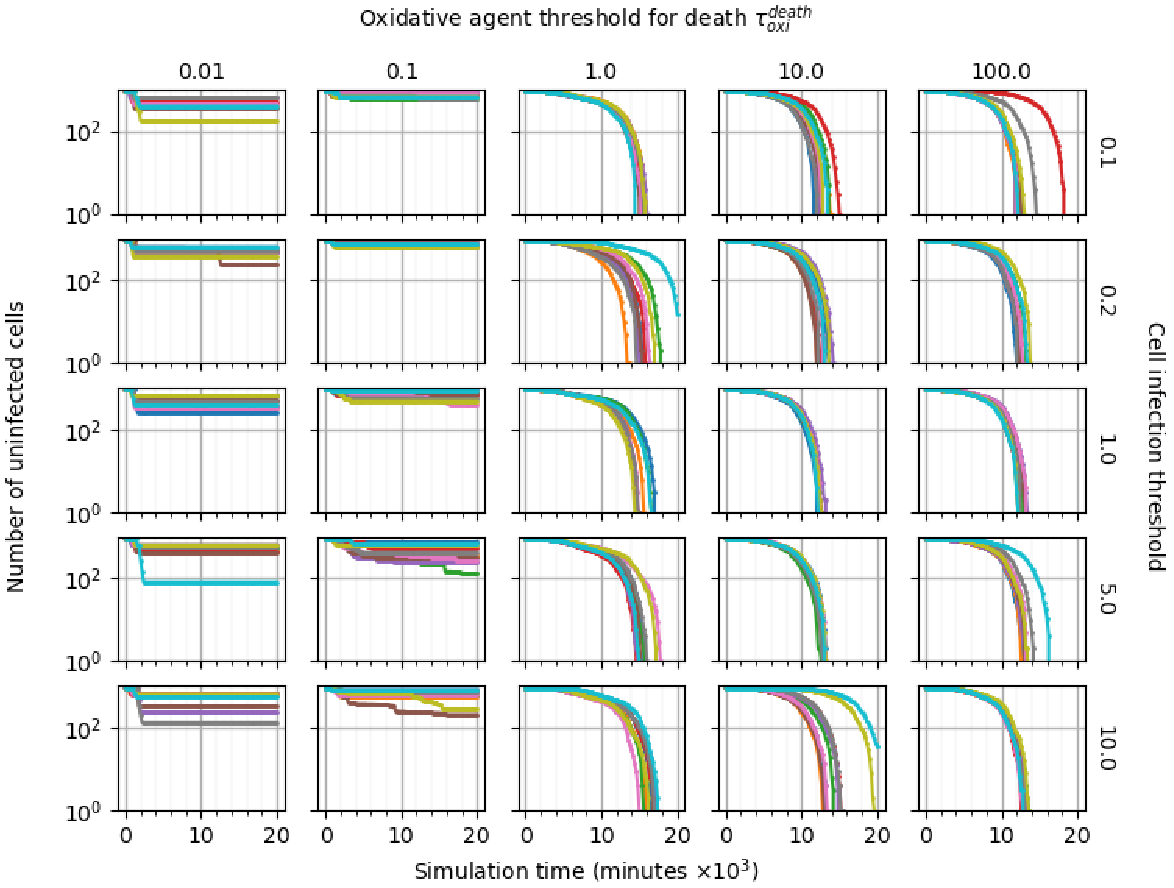

Fig S19. Pairwise parameter sweep of the oxidative agent threshold for death τ_{oxi}^{death} ($\times$ 0.01, $\times$ 0.1, $\times$ 1, $\times$ 10, $\times$ 100) and the infection threshold ($\times$ 0.1, $\times$ 0.2, $\times$ 1, $\times$ 5, $\times$ 10) around their baseline values, with ten simulation replicas per parameter set (all other parameters have their baseline values as given in Table 1). The number of uninfected epithelial cells for each simulation replica for each parameter set, plotted on a logarithmic scale, *vs* time displayed in minutes.

bioRxiv preprint doi: https://doi.org/10.1101/2020.04.27.064139. this version posted September 26, 2020. The copyright holder for this preprint (which was not certified by peer review) is the author/funder. It is made available under a CC-BY 4.0 International license.

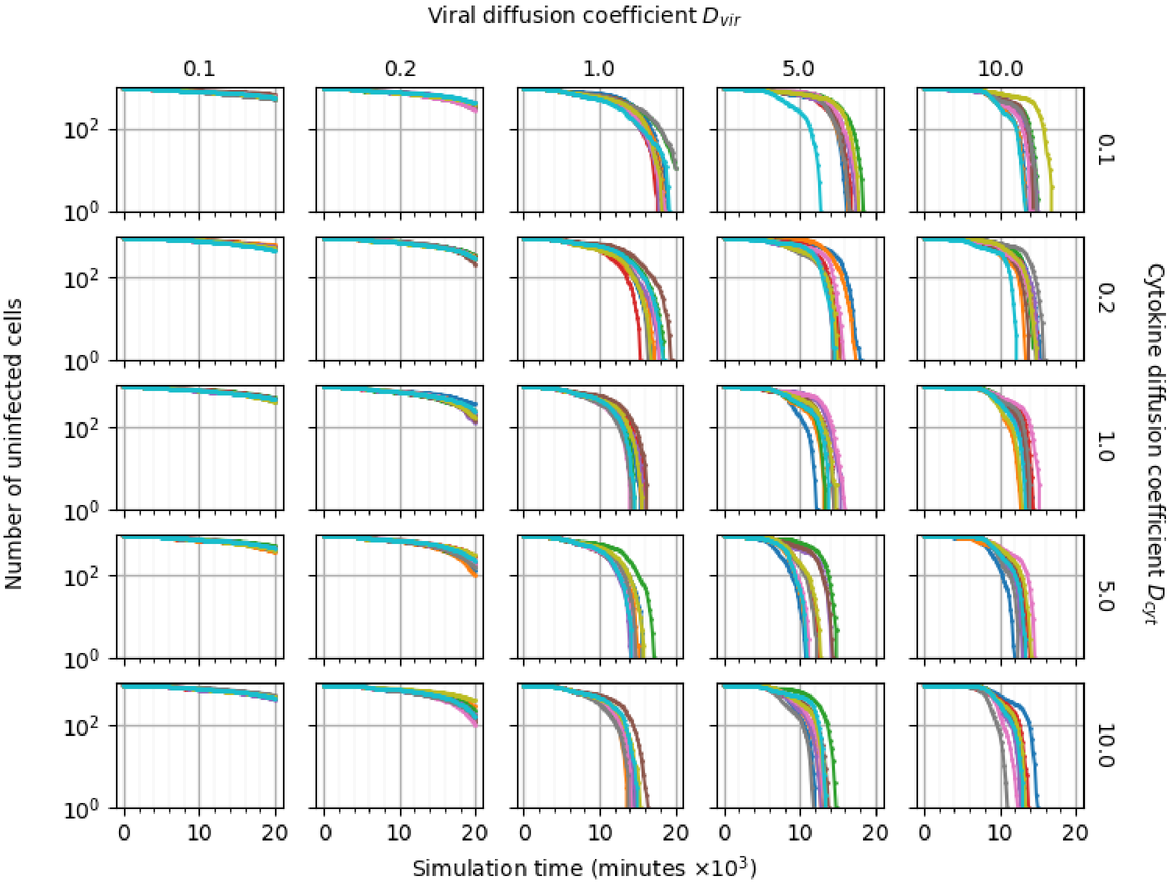

Fig S20. Pairwise parameter sweep of the viral diffusion coefficient D_{vir} and the cytokine diffusion coefficient D_{cyt} ($\times$ 0.1, $\times$ 0.2, $\times$ 1, $\times$ 5, $\times$ 10) around their baseline values, with ten simulation replicas per parameter set (all other parameters have their baseline values as given in Table 1).

The number of uninfected epithelial cells for each simulation replica for each parameter set, plotted on a logarithmic scale, *vs* time displayed in minutes.

bioRxiv preprint doi: https://doi.org/10.1101/2020.04.27.064139. this version posted September 26, 2020. The copyright holder for this preprint (which was not certified by peer review) is the author/funder. It is made available under a CC-BY 4.0 International license.

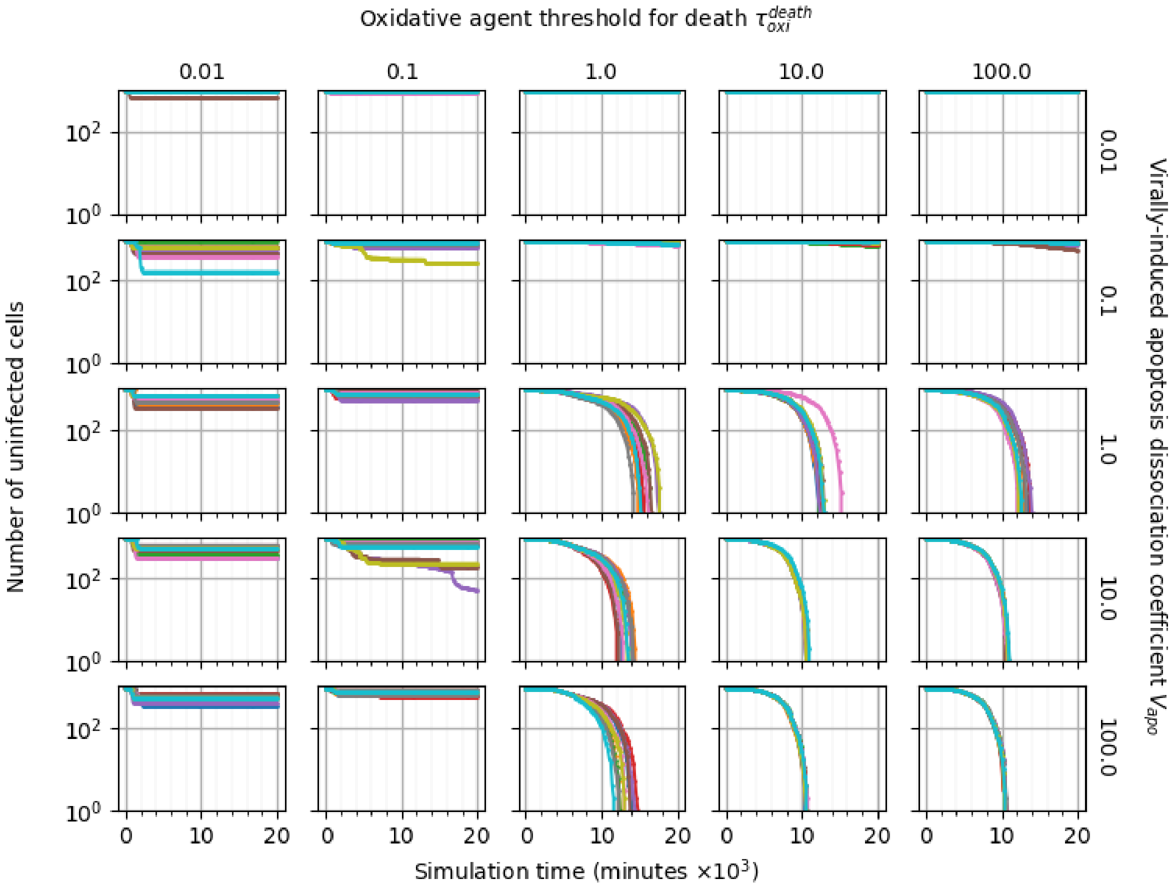

Fig S21. Pairwise parameter sweep of the oxidative agent threshold for death τ_{oxi}^{death} and the virally-induced apoptosis dissociation coefficient V_{apo} ($\times$ 0.01,$\times$ 0.1,$\times$ 1,$\times$ 10,$\times$ 100) around their baseline values, with ten simulation replicas per parameter set (all other parameters have their baseline values as given in Table 1). The number of uninfected epithelial cells for each simulation replica for each parameter set, plotted on a logarithmic scale, *vs* time displayed in minutes.

bioRxiv preprint doi: https://doi.org/10.1101/2020.04.27.064139. this version posted September 26, 2020. The copyright holder for this preprint (which was not certified by peer review) is the author/funder. It is made available under a CC-BY 4.0 International license.

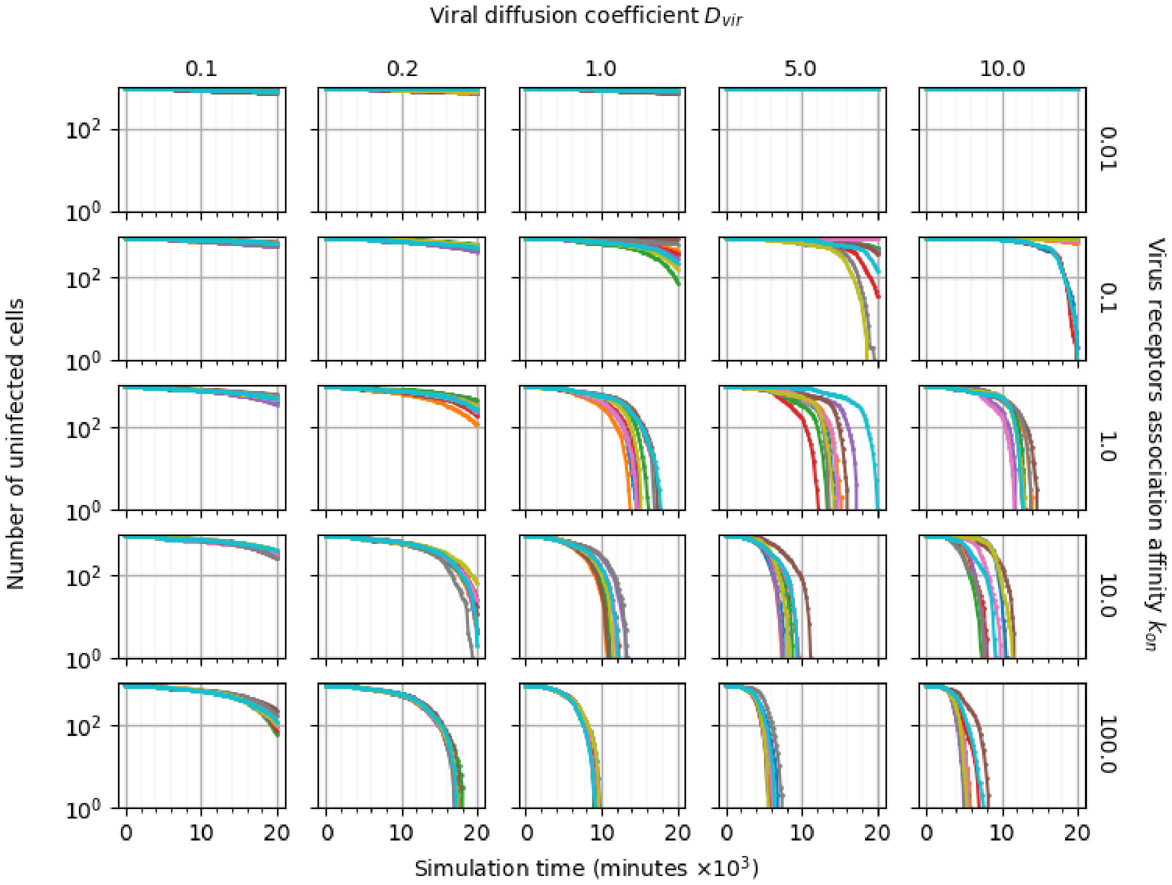

2007

2008 **Fig S22. Pairwise parameter sweep of the viral diffusion coefficient D_{vir} (× 0.1,× 0.2,× 1,× 5,× 10) and the**
2009 **virus-receptor association affinity k_{on} (× 0.01,× 0.1,× 1,× 10,× 100) around their baseline values, with ten**
2010 **simulation replicas per parameter set (all other parameters have their baseline values as given in Table 1).**
2011 The number of uninfected epithelial cells for each simulation replica for each parameter set, plotted on a logarithmic
2012 scale, *vs* time displayed in minutes.

2013

bioRxiv preprint doi: https://doi.org/10.1101/2020.04.27.064139. this version posted September 26, 2020. The copyright holder for this preprint (which was not certified by peer review) is the author/funder. It is made available under a CC-BY 4.0 International license.

A non-uniform epithelial sheet shows no significant effects in emergent dynamics of simulation outcomes

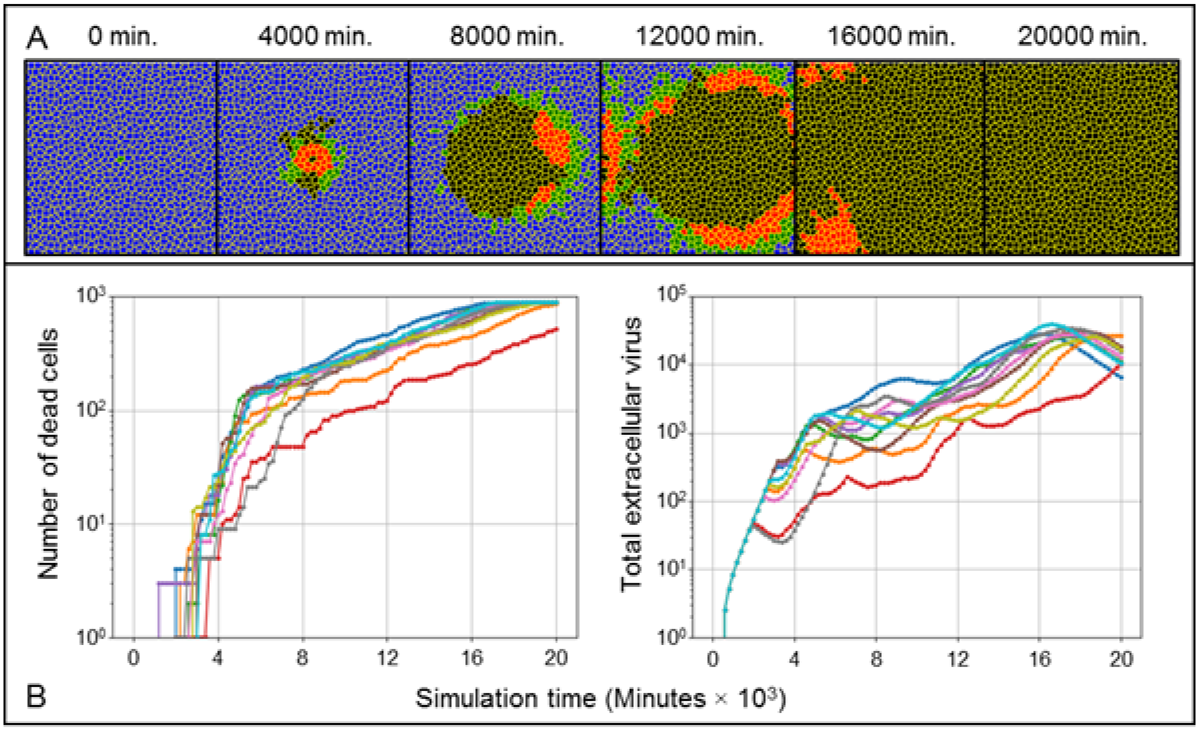

Fig S23. Simulation of viral infection using the baseline parameter set as in Fig 3 but with a non-uniform epithelial sheet.

(A) Widespread infection occurs with the same spatiotemporal features as in Fig 3A in a non-uniform epithelial sheet. (B) Ten simulation replicas with a non-uniform epithelial sheet showed no significant differences in transient metrics compared to simulations with a uniform epithelial sheet.

Integration of an explicit RNA synthesis model

The HCV model in [59] describes subgenomic replication in two compartments, namely the cytoplasm and vesicular membrane structures (VMS). Integration with the viral replication model described in *Quantitative model and implementation* requires the two modifications, one to the HCV model, and one to the viral replication model of the main framework, such that the viral genome taking part in genomic replication from (7) is

bioRxiv preprint doi: https://doi.org/10.1101/2020.04.27.064139. this version posted September 26, 2020. The copyright holder for this preprint (which was not certified by peer review) is the author/funder. It is made available under a CC-BY 4.0 International license.

a proxy for the cytoplasmic plus-strand RNA molecules of the HCV model. Both modifications are described here.

According to the HCV model, in the cytoplasm,

$$\frac{dR_P^{cyt}}{dt} = k_2 T_c + k_{Pout} R_P - k_I R_{ibo} R_P^{cyt} - k_{Pin} R_P^{cyt} - \mu_P^{cyt} R_P^{cyt} + n_{HCV} r_u U, \quad \text{(S1)}$$

$$\frac{dT_c}{dt} = k_I R_{ibo} R_P^{cyt} - k_2 T_c - \mu_{Tc} T_c, \quad \text{(S2)}$$

$$\frac{dP^{cyt}}{dt} = k_2 T_c - k_c P^{cyt}, \quad \text{(S3)}$$

$$\frac{dE^{cyt}}{dt} = k_c P^{cyt} - k_{Ein} E^{cyt} - \mu_E^{cyt} E^{cyt}, \quad \text{(S4)}$$

where R_P^{cyt} is the number of plus-strand HCV RNA molecules in the cytoplasm, T_c is the number of translation complexes in the cytoplasm, P^{cyt} is the number of HCV polyprotein molecules in the cytoplasm, E^{cyt} is the number of enzyme NS5B and other viral proteins needed for RNA synthesis in the cytoplasm, R_{ibo} is the number of host cell ribosomes ($R_{ibo} = R_{ibo}^{tot} - T_c$ for fixed total available ribosomes R_{ibo}^{tot}), and n_{HCV} relates R_P^{cyt} to unitless R. Simulations of the HCV model were performed as in [59] by initializing R_P^{cyt} with an initial nonzero value in the initially infected cell. In the case of a spatial context, where cells are infected at various times according to progression of infection and subsequent internalization events, subgenomic replication within a particular cell occurs due to internalization of virus by the cell (6). As such, the final term of (S1) was added during integration such that internalized virus acts as a source for R_P^{cyt}.

bioRxiv preprint doi: https://doi.org/10.1101/2020.04.27.064139. this version posted September 26, 2020. The copyright holder for this preprint (which was not certified by peer review) is the author/funder. It is made available under a CC-BY 4.0 International license.

Likewise, in the VMS,

$$\frac{dR_P}{dt} = -k_3 R_P E + k_{4p} R_{Ids} + k_{Pin} R_P^{cyt} - (k_{Pout} + \mu_P) R_P, \quad \text{(S5)}$$

$$\frac{dR_{ds}}{dt} = k_{4m} R_{Ip} + k_{4p} R_{Ids} - k_5 R_{ds} E - \mu_{ds} R_{ds}, \quad \text{(S6)}$$

$$\frac{dE}{dt} = k_{Ein} E^{cyt} + k_{4m} R_{Ip} + k_{4p} R_{Ids} - k_3 R_P E - k_5 R_{ds} E - \mu_E E, \quad \text{(S7)}$$

$$\frac{dR_{Ip}}{dt} = k_3 R_P E - k_{4m} R_{Ip} - \mu_{Ip} R_{Ip}, \quad \text{(S8)}$$

$$\frac{dR_{Ids}}{dt} = k_5 R_{ds} E - k_{4p} R_{Ids} - \mu_{Ids} R_{Ids}, \quad \text{(S9)}$$

where R_P is the number of plus-strand RNA in the VMS, R_{ds} is the number of dsRNA in the VMS, E is the number of HCV polymerase complexes in the VMS, R_{Ip} is the number of plus-strand RNA replicative intermediate complexes in the VMS and R_{Ids} is the number of plus-strand dsRNA replicative intermediate complexes in the VMS.

Having selected R_P^{cyt} and R as the shared biological object of the two models, mass action $R \rightarrow P$ of the viral replication model of the main framework requires modification. We assume that decay of R_P^{cyt} described in the HCV model leads to production of P through intermediate processes. The viral replication model of the main framework ((6)–(10)) then takes the modified form,

$$\frac{dU}{dt} = Uptake - r_u U, \quad \text{(S10)}$$

$$n_{HCV} R = R_P^{cyt}, \quad \text{(S11)}$$

bioRxiv preprint doi: https://doi.org/10.1101/2020.04.27.064139. this version posted September 26, 2020. The copyright holder for this preprint (which was not certified by peer review) is the author/funder. It is made available under a CC-BY 4.0 International license.

$$\frac{dP}{dt} = r_t{}'R - r_pP, \tag{S12}$$

$$\frac{dA}{dt} = r_pP - Release, \tag{S13}$$

where $r_t{}'$ is the rate of production of P per unit of R associated with decay of R_P^{cyt}. Note that without the introduction of additional decay to the HCV model equations, the integrated form of the HCV model effectively acts the same as its original form within each cell, subject to the cellular and spatial aspects of internalization events. All parameters of the integrated HCV model in simulations shown in *Particularization to hepatitis C virus by integration of an explicit RNA synthesis model* were taken from [59] (Table S2).

Table S2. Parameter values of integrated HCV model.

Simulation parameters	Value
T_c formation rate k_1	4800 molecule^{-1} min.$^{-1}$
Nascent polyprotein cleavage rate k_2	6000 min.$^{-1}$
Viral polyprotein cleavage rate k_c	36 min.$^{-1}$
R_P^{cyt} transport rate into cytoplasm k_{Pin}	12 min.$^{-1}$
R_P transport rate into VMS k_{Pout}	12 min.$^{-1}$
E^{cyt} transport rate in VMS k_{Ein}	7.8×10-4 min.$^{-1}$
R_{Ip} formation rate k_3	1.2 molecule^{-1} min.$^{-1}$
R_P synthesis rate k_{4p}	102 min.$^{-1}$
R_{ds} synthesis rate k_{4m}	102 min.$^{-1}$
R_{Ids} formation rate k_5	240 min.$^{-1}$
R_P^{cyt} degradation rate μ_P^{cyt}	600 min.$^{-1}$
R_P degradation rate μ_P	4.2 min.$^{-1}$
R_{ds} degradation rate μ_{ds}	3.6 min.$^{-1}$

bioRxiv preprint doi: https://doi.org/10.1101/2020.04.27.064139. this version posted September 26, 2020. The copyright holder for this preprint (which was not certified by peer review) is the author/funder. It is made available under a CC-BY 4.0 International license.

R_{Ip} degradation rate μ_{Ip}	2.4 min.$^{-1}$
R_{Ids} degradation rate μ_{Ids}	7.8 min.$^{-1}$
T_c degradation rate μ_{Tc}	0.9 min.$^{-1}$
E degradation rate μ_E	2.4 min.$^{-1}$
E^{cyt} degradation rate μ_E^{cyt}	3.6 min.$^{-1}$
Total number of available ribosomes R_{ibo}^{tot}	700
RNA conversion factor n_{HCV}	100 molecule R^{-1}
P production rate r_t'	2.5 min.$^{-1}$ R^{-1}

Collaborative viral infection modeling environment

Given the immense amount of complexity associated with viral infection, supporting collaborative, independent, concurrent, and even conflicting, model development is critical to building an informative and predictive multiscale model of viral infection. As such, the simulation architecture developed for the CompuCell3D implementation, as demonstrated in *Model extensions*, supports development, deployment and distribution of add-on modules following the Python programming language design principles and practices of extensibility and modularity. This architecture exploits the architecture of CompuCell3D itself, specifically, that model implementation in CompuCell3D consists of designing a set of Python classes called "steppables", each of which is imported into CompuCell3D and simulated (via exactly two simple lines of Python code per steppable, see *Deploying a model extension in CompuCell3D*). Each steppable typically implements a particular model, function, or feature (*e.g.*, viral internalization, data post-processing and exporting), and provides instructions to CompuCell3D about what to do during each simulation step along with the core simulation engine (*e.g.*, implementing

bioRxiv preprint doi: https://doi.org/10.1101/2020.04.27.064139. this version posted September 26, 2020. The copyright holder for this preprint (which was not certified by peer review) is the author/funder. It is made available under a CC-BY 4.0 International license.

the Cellular Potts Model), as well as what to do before and after simulation, through a simple interface (*e.g.*, procedures to perform by a steppable during each simulation step are described in a function “step” in the steppable class definition). This approach is particularly well suited for supporting collaborative, independent, and concurrent model development because model specification of a particular simulation in CompuCell3D consists of selecting and loading a particular set of steppables, each of which can be specified in separate Python scripts and packaged in uniquely named directories, developed by collaborating or independent and otherwise disconnected research groups, and intended to model specific biological phenomena. Furthermore, specification of model implementations using Python classes also enables development of model extensions from existing modules (whether from the main framework or an add-on module) using basic Python class inheritance functionality (where class definitions can be constructed from other class definitions and subsequently modified, see *Extending a model in CompuCell3D*).

We envision a community of modelers much like the community of Python developers, which develops Python packages, called “modules”, that can be publicly distributed and imported into software using a simple, one-line Python command (*e.g.*, import MyModule). As such, we have built into the CompuCell3D implementation used in this work a location for storing a library of add-on modules, as well as supporting architecture to facilitate development and deployment of add-on modules. Furthermore, along with making the simulation framework publicly available online as described in *Downloading and running the simulation*, the online repository also hosts this library of add-on modules as part of the standard download package, which we continue to develop

bioRxiv preprint doi: https://doi.org/10.1101/2020.04.27.064139. this version posted September 26, 2020. The copyright holder for this preprint (which was not certified by peer review) is the author/funder. It is made available under a CC-BY 4.0 International license.

and maintain, and for which we are currently developing standards (*e.g.*, standard documentation) and supporting tools (*e.g.*, documentation generators). We welcome usage by, and contributions from, all interested groups, and provide a basic overview of deploying and developing model extensions in the remaining discussion of this section.

Deploying a model extension in CompuCell3D. As in any typical CompuCell3D model specification, one script of the simulation files shown in Fig S13, Simulation/ViralInfectionVTM.py, imports all modules of the main framework and loads them into CompuCell3D for simulation. The directory “Simulation” contains all source code of the main framework, while an additional directory “Models” is dedicated to storing source code of the add-on module library. Each add-on module is an importable Python module stored in its own, uniquely named subdirectory (*e.g.*, Models/IUBIOCAddons). Code Snippet S1 shows a section of the contents of Simulation/ViralInfectionVTM.py for a simulation using the Simple Recovery model described in *An extensible framework architecture enables the inclusion of tissue recovery*.

```
from ViralInfectionVTMSteppables import ViralInternalizationSteppable
CompuCellSetup.register_steppable(steppable=ViralInternalizationSteppable(frequency=1))

from Models.RecoverySimple.RecoverySteppables import SimpleRecoverySteppable
CompuCellSetup.register_steppable(steppable=SimpleRecoverySteppable(frequency=1))
```

Code Snippet S1. Select import and load commands from Simulation/ViralInfectionVTM.py.

bioRxiv preprint doi: https://doi.org/10.1101/2020.04.27.064139. this version posted September 26, 2020. The copyright holder for this preprint (which was not certified by peer review) is the author/funder. It is made available under a CC-BY 4.0 International license.

Lines 1 and 2 in Code Snippet S1 import and load the steppable "ViralInternalizationSteppable" that implements the internalization model described in *E1 - Viral internalization* from Simulation/ViralInfectionVTMSteppables.py. Lines 4 and 5 show that not much is different concerning loading and importing add-on modules. The steppable "SimpleRecoverySteppable" implements the Simple Recovery Model, and is defined in Models/RecoverySimple/RecoverySteppables.py. The only difference between importing and loading a module from the main framework or add-on module library is specifying the location of the Python script containing the steppable to be deployed in a simulation. This way, two model modules can define steppables in Python scripts of the same name without overwriting each other (*e.g.*, Models/GroupX/Steppables.py or Models/GroupY/Steppables.py). The only necessarily unique aspect of a particular model module is the name of its containing directory (*e.g.*, the directory Models/GroupX or Models/GroupY). This scheme isolates model-specific development to the directory in which the add-on model is defined, and modularizes the overall simulation framework into *shareable*, *interchangeable* model components. Furthermore, since development of add-on modules is isolated to a uniquely named directory, the framework promotes concurrent development and implementation of unrelated or even competing models.

Developing a Model Extension in CompuCell3D. Developing a model extension is as simple as the typical CompuCell3D model implementation procedure of developing steppables in Python using the CompuCell3D steppable class "SteppableBasePy" (see

bioRxiv preprint doi: https://doi.org/10.1101/2020.04.27.064139. this version posted September 26, 2020. The copyright holder for this preprint (which was not certified by peer review) is the author/funder. It is made available under a CC-BY 4.0 International license.

Extending a model in CompuCell3D for discussion of Python class inheritance). Code Snippet S2 shows the application programming interface (API) and select code from the Python script Models/RecoverySimple/RecoverySteppables.py in the add-on module library that implements the Simple Recovery model described in *An extensible framework architecture enables the inclusion of tissue recovery*.

bioRxiv preprint doi: https://doi.org/10.1101/2020.04.27.064139. this version posted September 26, 2020. The copyright holder for this preprint (which was not certified by peer review) is the author/funder. It is made available under a CC-BY 4.0 International license.

```
1    import sys
2    import os
3    # Import from simulation environment
4    sys.path.append(os.environ["ViralInfectionVTM"])
5    # Import parameter values from RecoveryInputs.py and other stuff
6    from .RecoveryInputs import *
7    from Simulation.ViralInfectionVTMModelInputs import s_to_mcs
8    import random
9    from cc3d.core.PySteppables import *
10
11   class SimpleRecoverySteppable(SteppableBasePy):
12       def __init__(self, frequency=1):
13           """
14           Initialize recovery steppable
15           """
16
17       def start(self):
18           """
19           Share self with framework
20           """
21
22       def step(self, mcs):
23           """
24           Perform recovery test in each dead cell
25           """
26
27       def recover_cell(self, _cell):
28           """
29           Implement recovery
30           """
31
32       def cell_recovers(self, _cell) -> bool:
33           """
34           Test for simple recovery in a cell
35           """
36           return random.random() < recovery_rate * s_to_mcs
```

Code Snippet S2. API for the steppable implementing the Simple Recovery model, derived from Models/RecoverySimple/RecoverySteppables.py.

The exact code of the implementation is shown for the steppable function “cell_recovers” (Lines 32-36).

bioRxiv preprint doi: https://doi.org/10.1101/2020.04.27.064139. this version posted September 26, 2020. The copyright holder for this preprint (which was not certified by peer review) is the author/funder. It is made available under a CC-BY 4.0 International license.

Lines 1-4 in Code Snippet S2 add the directory containing both the main framework and add-on modules library directories using the environment variable "ViralInfectionVTM", which makes both available to any module loaded into CompuCell3D from Simulation/ViralInfectionVTM.py, whether directly or indirectly (*e.g.*, when Simulation/ViralInfectionVTM.py imports module "A" that imports module "B"). Line 6 imports parameter values of this module defined in Models/RecoverySimple/RecoveryInputs.py, while Line 7 imports a parameter value from the main framework for use in calculations. Line 8 imports the Python standard module "random" for generating random numbers, which, like the rest of the Python standard library and many others, is distributed with CompuCell3D. Line 9 imports everything from the CompuCell3D module "PySteppables", which contains the available Python classes for Python model implementation in CompuCell3D.

Line 11 in Code Snippet S2 begins the definition of the Simple Recovery model steppable, "SimpleRecoverySteppable". SimpleRecoverySteppable inherits from "SteppableBasePy", a steppable class defined in the PySteppables module. Its first three functions, "__init__", "start", and "step", are all functions of the CompuCell3D steppable interface. "__init__" defines the procedures to be performed for initializing the steppable. "start" defines the procedures to be performed after CompuCell3D has initialized but before simulation begins (in the case of this steppable, sharing a reference to itself with the rest of the framework). "step" defines the procedures to be performed during each simulation step (in this case of this steppable, evaluating recovery in each dead cell and

bioRxiv preprint doi: https://doi.org/10.1101/2020.04.27.064139. this version posted September 26, 2020. The copyright holder for this preprint (which was not certified by peer review) is the author/funder. It is made available under a CC-BY 4.0 International license.

executing recovery when it occurs). The final two functions are specific to this steppable. The first, "recover_cell", performs the necessary procedures associated with recovery on a cell when given one as an argument (*i.e.*, "_cell"). The second, "cell_recovers", evaluates whether or not a particular cell is recovered. It should be noted that deployment of the SimpleRecoverySteppable class is not limited to usage directly in CompuCell3D as a simulated steppable. Rather, like the importing of a parameter value from the main framework in Line 7 of Code Snippet S2, the SimpleRecoverySteppable class can be imported into other modules for other purposes, like performing recovery of a dead cell but due to an alternative recovery criterion. The following section describes an example of such functionality.

Extending a model in CompuCell3D. Like any other Python class, steppables (and other code) defined in one model module can be extended by, or integrated into, other modules, such that the components of the overall simulation framework are not only interchangeable and shareable, but also extensible. In the previous section, Code Snippet S2 demonstrated the ability to import a parameter value (*i.e.*, "s_to_mcs", Line 7) from the main framework for usage in an add-on module. The same can be done for integrating modules (whether from the main framework or add-on library) into other add-on modules, as well as for extending model modules using Python class inheritance. Python class inheritance enables the construction of classes from the definition of other classes, such that functionality and interfaces defined by one class can be employed, selectively adapted, and extended, by subsequent classes that inherit from it. Any inheriting class, called a "derived class", can replace (*i.e.*, "overwrite") the executed

bioRxiv preprint doi: https://doi.org/10.1101/2020.04.27.064139. this version posted September 26, 2020. The copyright holder for this preprint (which was not certified by peer review) is the author/funder. It is made available under a CC-BY 4.0 International license.

code of a function in the definition of an inherited class, called a “base class”, if the derived class defines a function with the same name and arguments. All inherited functions that are not overwritten by a derived class are the same.

Simulation results of the Neighbor Recovery model in *An extensible framework architecture enables the inclusion of tissue recovery* demonstrate deployment using the framework capability of constructing add-on modules from other add-on modules. Computationally, nearly all aspects of the Recovery Model are the same as the Simple Recovery model (*e.g.*, test for recovery in every cell during each simulation step, implement recovery when it occurs). The only difference between the two models is the criterion by which recovery of a dead cell is evaluated, making the implementation of the Neighbor Recovery model a strong candidate for exploiting Python class inheritance, as demonstrated in Code Snippet S3.

```
import sys
import os
# Inherit from Simple Recovery model
sys.path.append(os.environ["ViralInfectionVTM"])
from Models.RecoverySimple.RecoverySteppables import SimpleRecoverySteppable

class NeighborRecoverySteppable(SimpleRecoverySteppable):
    def __init__(self, frequency=1):
        super().__init__(frequency)

    def cell_recovers(self, _cell) -> bool:
        """
        Test for neighbor-dependent recovery in a cell
        """
```

bioRxiv preprint doi: https://doi.org/10.1101/2020.04.27.064139. this version posted September 26, 2020. The copyright holder for this preprint (which was not certified by peer review) is the author/funder. It is made available under a CC-BY 4.0 International license.

Code Snippet S3. API for the steppable implementing the Simple Recovery model, derived from Models/RecoveryNeighbor/RecoverySteppables.py.

As in Code Snippet S2, Lines 1-4 of Code Snippet S3 makes available the entire framework, while Line 5 imports the Simple Recovery model steppable definition for extension. Line 7 begins the definition of the steppable "NeighborRecoverySteppable" that implements the Neighbor Recovery model by inheriting from the class definition for the Simple Recovery model "SimpleRecoverySteppable". Since all functionality of the Neighbor Recovery steppable is to be the same as the Simple Recovery steppable except for the recovery criterion, Lines 8-9 initialize the Neighbor Recovery steppable exactly the same as the Simple Recovery steppable. Lines 11-14 begin overwriting the definition of the recovery criterion from Simple Recovery according to the Neighbor Recovery model. Since no other functions of the Simple Recovery steppable are overwritten, they are then exactly the same for the Neighbor Recovery steppable. Furthermore, since the signature of the function that implements the recovery criterion (*i.e.*, "cell_recovers(self, _cell)") has the exact same name and arguments in both steppables (*i.e.*, the function "cell_recovers" is overwritten by NeighborRecoverySteppable), they can be used in the exact same way by other modules (*e.g.*, a variable "my_recovery_steppable", whether an instance of SimpleRecoverySteppable or NeighborRecoverySteppable, receives and returns the same type of information). The only difference in behavior between using one or the other is the potential outcome of asking either recovery steppable whether or not a particular dead cell recovers, about which the two models will often disagree.
